# Patient Activeness During Online Medical Consultation in China: Multilevel Analysis

**DOI:** 10.2196/35557

**Published:** 2022-05-27

**Authors:** Bolin Cao, Wensen Huang, Naipeng Chao, Guang Yang, Ningzheng Luo

**Affiliations:** 1 School of Media and Communication Shenzhen University Shenzhen China; 2 Health 160 Shenzhen China

**Keywords:** patient, physician, online medical consultation, patient activeness

## Abstract

**Background:**

Online medical consultation is an important complementary approach to offline health care services. It not only increases patients’ accessibility to medical care, but also encourages patients to actively participate in consultation, which can result in higher shared decision making, patient satisfaction, and treatment adherence.

**Objective:**

This study aims to explore multilevel factors that influence patient activeness in online medical consultations.

**Methods:**

A data set comprising 40,505 patients from 300 physicians in 10 specialties was included for multilevel analysis. Patient activeness score (PAS) was calculated based on the frequency and the proportion of patient discourses to the total frequency of doctor-patient interactions. Intraclass correlation coefficients were calculated to identify between-group variations, and the final multilevel regression model included patient- and physician-level factors.

**Results:**

Patients were not equally active in online medical consultations, with PASs varying from 0 to 125.73. Patient characteristics, consultation behavioral attributes, and physician professional characteristics constitute 3 dimensions that are associated with patient activeness. Specifically, young and female patients participated more actively. Patients’ waiting times online (*β*=–.17; *P*<.001) for physician responses were negatively correlated with activeness, whereas patients’ initiation of conversation (*β*=.83; *P*<.001) and patient consultation cost (*β*=.52; *P*<.001) in online medical consultation were positively correlated. Physicians’ online consultation volumes (*β*=–.10; *P*=.01) were negatively associated with patient activeness, whereas physician online consultation fee (*β*=.03; *P*=.01) was positively associated. The interaction effects between patient- and physician-level factors were also identified.

**Conclusions:**

Patient activeness in online medical consultation requires more scholarly attention. Patient activeness is likely to be enhanced by reducing patients’ waiting times and encouraging patients’ initiation of conversation in online medical consultation. The findings have practical implications for patient-centered care and the improvement of online medical consultation services.

## Introduction

Online medical consultation is increasingly being chosen by patients as an alternative to traditional health care services. It is an electronic format for doctor-patient interaction, connecting both parties of medical services through text, pictures, and videos [1-3]. The development of online medical consultation is a result of rapid technological advancement and broad market demand [2,4,5]. Specifically, online medical consultation has been emphasized during the COVID-19 pandemic to prevent cross-infection or to implement social distancing rules as encouraged by many governments [6]. Given its advantages in removing temporal and spatial barriers and documenting the medication process, online medical consultation is sometimes more appealing to patients than offline medical encounters [2,7]. According to the global market estimate reports, the market share of online medical consultation was US $3.9 billion in 2020 and is estimated to reach US $16.0 billion by 2026 [8].

Although online medical consultation has been widely recognized for its potential, its strengths remain underappreciated. An overlooked benefit is its encouragement of patient activeness. Patient activeness is an emerging term to describe patients’ active participation in managing their health and wellness. Patients often participate in their medical visits by seeking and providing information, asserting their preferences or opinions, and expressing their concerns [9]. Through these formats, patients can gain more knowledge and control over their health, leading to improved health outcomes [7]. Patient activeness has also been found to have significant impacts on shared decision making, patient satisfaction, and treatment adherence [10-12].

Following the patient-centered approach, online medical consultation may empower patients with more opportunities to actively participate in the health care–seeking process. First, patients can express themselves extensively without feeling rushed in online medical consultation. Patients can deliver unlimited messages in the text format without being interrupted by the physician or another patient [1,13]. Second, patients who feel tense and shy in the offline setting might be freer to disclose themselves, given that the absence of a visual audience may allow them to let go of their sense of being watched or embarrassed [13]. Third, patients can access the internet or other resources for additional information in online medical consultation, enhancing their ability to participate in online medical conversations with their physicians [2,7]. The internet can help patients bridge the information gap with their physicians and ask more meaningful questions. As a result of these features of online medical consultation, the online doctor-patient communication process can be transformed from paternalism to partnership, encouraging patient activeness [14].

Traditionally, health providers play a paternalistic role in China, only providing information and treatments they consider necessary and useful for patient recovery [15]. This leads to patients being viewed as passive, rarely offering an opinion, and not participating in medical decision making [16]. Most offline consultations last for less than 5 minutes, and the patient participates only minimally during the consultation [17,18]. Like many other countries, China has seen an increase in the demand for online medical care. According to a report published by consultancy iiMedia Research [4], the market for online medical care in China was US $8.41 billion in 2019. Chinese internet users who use online medical services have increased threefold from 2015 to 2020, with a total of 661 million users. Most online medical consultation platforms offer text consultation, telephone consultation, and video consultation. Telephone consultations and video consultations are both synchronous, while text consultations are asynchronous. However, the most common form of online consultation among patients is text consultation due to several reasons, including the fact that most physicians conduct consultations during their spare time, scheduling appointments for a telephone or video consultation is challenging, and the cost of telephone or video consultations are high [18].

This study aims to investigate factors that influence patient activeness during online medical consultation in China. Despite its popularity, online medical consultation remains in its infancy in China [12]. Patients’ active participation in online medical consultation could increase their preference and satisfaction with the service. As online medical consultation involves interpersonal and environmental dynamics [19,20], this study examines the multilevel factors that influence patient participation in online consultation.

## Methods

### Data Collection and Research Design

Data for this study were collected from a third-party online medical consultation platform, Health 160, a pioneering online medical service provider. Established in 2005, it has served over 170 million patients associated with 610,000 physicians. Anonymous and deidentified consultation data were obtained from this platform, complying with the users’ consent to privacy policies. A multistage sampling scheme was conducted, where the top 10 disease specialties were selected on the basis of their popularity first, and then 30 physicians were selected at random within each disease specialty. Between May 2019 and May 2020, these 300 physicians’ consultation records were retrieved, yielding 57,378 consultations from 40,505 patients. Given that some patients had more than 1 order for consultation in the sample, the first record for a specific patient was utilized.

A hierarchical data structure was used in this study, with patients nested within physicians within disease specialties. The 40,505 consultation records were considered as the first layer of the research, which included patient characteristics and consultation behavior data, and 300 physician-level data as the second layer of the research.

### Ethics Approval

The study protocol was approved by the institutional review board at Shenzhen University (approval number 2020028).

### Measure

Patient activeness was measured by the patient activeness score (PAS), which expresses the proportion of patient discourses to the total frequency of doctor-patient communication in online medical consultation. The formula of calculating PAS is as follows:

PAS = n × [n/(n+m)]

where n and m refer to the numbers of patient discourses and physician expressions for each consultation, respectively. Given the highly skewed distribution of PAS from 0 to 125.73, a logarithmical transformation was used for each score: f(x) = ln(x+0.01), which reduced the variability of scores to [–4.61 to 4.83], denoted as logPAS.

### First-Level Variables

Patient demographics and the consultation behavioral characteristics constitute the first-level factors of analysis. Patient demographics include gender and age, and the consultation behavioral characteristics include patients’ waiting time for response, patients’ initiation of consultation, and patient cost for consultation service. Patients’ waiting time for response refers to how long they waited for the doctor to respond after a consultation has been initiated. The time was primarily measured in seconds and then converted to log10: f(x) = log10(x). Patients’ initiation of consultation was dummy coded as whether the conversation was started by the patient or the physician (patient = yes, physician = no). In addition, the online consultation services on the Health 160 platform are generally paid, but some free consultation services are provided to promote service use, facilitate user trials, and aid in the prevention and control of the COVID-19 epidemic during the time the data were collected. Thus, the patient cost for consultation service was also dummy coded as paid or free.

### Second-Level Variables

In our study design, physician-level factors were considered second-level variables to investigate the effect of group heterogeneity on patient activeness. These factors included physician demographics and professional characteristics. The demographic variables included physician age (in years) and gender (1 = female, 0 = male). Their professional characteristics were indicated by their online consultation volumes and fees. Online consultation volume refers to the number of patients who have consulted the physician online. Given that the online consultation volumes ranged from 3 to 3916, they were log-transformed to values with a base of 10 and the range was 0.48-3.59. The consultation fee was recoded as an ordinal variable, ranging from 0 (0 CNY) to 6 (over 50 CNY [US $7.72]).

### Third-Level Variables

Specialty-related factors were deemed as the third-level variable. The 10 different specificities were coded as categorical variables for analysis. The descriptive statistics of first-, second-, and third-level variables are shown in Table 1.

### Statistical Analysis

Multilevel regression analysis was used to analyze the nested study design. Generally, multilevel regression analysis is considered convenient for modeling the possible contributions of contextual factors at higher levels. An initial analysis comprising a 3-level model that incorporated patient (Level 1), physician (Level 2), and disease specialty (Level 3) characteristics was first conducted. To identify whether the higher levels were critical to explaining the data, the intraclass correlation coefficient (ICC) was estimated (denoted as ρ), an indication of how much variation in the outcome variable can be explained by between-group variation. The ICC number ranges from 0 to 1, with higher values indicating a greater variance between groups. Statisticians suggested that a variance of 0.059 might be used as an experience criterion to determine whether the between-group difference was large enough to be regarded as above average [21]. At the specialty level, the ICC was evaluated to be 0.018, indicating a low level of clustering effect within disease specialties in which physicians were similar. About 13% of the variation was attributed to variation at the physician level (ie, ICC ρ = [0.017 + 0.105]/[0.017 + 0.105 + 0.848] = 0.126). Thus, a 2-level analysis was applied, ignoring the specialty level, with patients nested within physicians.

In our specific analysis, the effect of each predictor on the outcome variable was first analyzed using univariate regression. Second, 2-level predictors were included successively in the nested models, testing the relative contribution of each with multiple regression analysis. For each independent variable, the variance inflation factors were found to be below 1.80, indicating that the collinearity between independent variables could safely be ignored without experiencing multicollinearity problems.

In addition, several cross-level interactions were identified between physician- and patient-level factors that might influence patient activeness. For instance, patients’ waiting time for response was supposed to be related to physician online consultation volumes, patients’ initiation of consultation was supposed to be related to physician gender, and patient cost for consultation service was supposed to be related to physician online consultation fee. During the interaction analysis, the centering of the explanatory variables is advantageous when a multilevel model contains interactions, given that it provides a clear interpretation of interaction terms and facilitates computation and convergence [22]. In this study, a grand mean centering, subtracting the mean from all values, was performed for each variable involved in the interactions. All statistical analyses were performed using R, version 3.6.

## Results

### Sample Characteristics

Of the 40,505 patients included in this study, there are twice as many female patients (n=28,057, 69.27%) as male patients (n=12,448, 30.73%). The median (IQR) proportion score of patient discourse during consultation [*n*/(*n*+*m*)] was 0.54 (0.50-0.66). The frequency of patient discourse (*n*) during consultation ranged from 0 to 178, and the frequency of patient and physician discourses (*n*+*m*) in total during consultation ranged from 1 to 227.

Among the 300 physicians, there existed slightly more females (n=163, 54%) than males (n=137, 46%). One-third of physicians were attending physicians (97/300, 32.33%), and nearly two-thirds held the higher management titles of chief physicians (81/300, 27.00%) and deputy chief physicians (104/300, 34.67%). The majority of consultations (34,024/40,505, 84%) were initiated by physicians. The median (IQR) patients’ waiting time for response was 7350 (1486-23,974) seconds, which is roughly 2 hours. Approximately one-fourth (9820/40,505, 24.4%) of patients received free online consultation. Of the 300 physicians, 19.00% (n=57) did not charge at all and the majority (n=213) did not charge over 50 CNY (US $7.85), whereas 10% (n=30) charged over 50 CNY (US $7.85). As of May 2020, the median (IQR) level of physicians’ online consultation volume reached 94.5 (43-211.75) patients (Table 1).

**Table 1:** Characteristics of first-, second-, and third-level variables.

Characteristics						Values
**Patient-level characteristics (N=40,505)**						
	**Patient demographics**					
		Age, median (IQR)			27 (11-33)	
	**Gender, n (%)**					
		Male			12,448 (30.73)	
		Female			28,057 (69.27)	
	**Patient consultation behavioral characteristics**					
		Patients’ waiting time for response (seconds), median (IQR)			7350 (1486-23,974)	
	**Patients’ initiation of consultation, n (%)**					
		Yes			6318 (15.60)	
		No			34,187 (84.40)	
	**Patient cost for consultation, n (%)**					
		Paid			30,685 (75.76)	
		Free			9820 (24.24)	
**Physician-level characteristics (N=300)**						
	**Physician demographics**					
		Age, median (IQR)			44 (37-51)	
		**Gender, n (%)**				
			Male	137 (45.67)		
			Female	163 (54.33)		
		**Professional title, n (%)**				
			Chief physician	81 (27.00)		
			Deputy chief physician	104 (34.67)		
			Attending physician	97 (32.33)		
			Other	18 (6.00)		
		**Physician professional characteristics**				
			Physician online consultation volume, median (IQR)	94.5 (43-211.75)		
		**Physician online consultation fee (CNY), n (%)^a^**				
			0	57 (19.00)		
			1-10	12 (4.00)		
			11-20	94 (31.33)		
			21-30	52 (17.33)		
			31-40	12 (4.00)		
			41-50	43 (14.33)		
			≥51	30 (10.00)		
		**Disease specialties (N=40,505), n (%)**				
			Dermatology	7975 (19.69)		
			Gynecology	6840 (16.89)		
			Pediatrics	5017 (12.39)		
			Endocrinology	3982 (9.83)		
			Traditional Chinese Medicine	3942 (9.73)		
			Obstetrics	3263 (8.06)		
			Urology	2918 (7.20)		
			Stomatology	2562 (6.33)		
			Psychiatric	2516 (6.21)		
			General surgery	1490 (3.68)		

^a^CNY = US $0.16.

### Factors Associated With Patient Activeness During Online Medical Consultation

The correlation matrix of physician- and patient-level variables and their univariate effects on logPAS is shown in Table 2. No strong linear correlations existed between the predictor variables, given that all the Pearson correlation coefficients were between –0.1 and 0.5. In the bivariate models, the effects of almost all factors were significant (*P*<.001), except for physician age. Thus, the next step for multilevel regression included them all as predictors. Our model included the physician’s age as a control variable, given that it was associated with the use of online medical consultation service in previous studies [22], although it failed to exert a significant effect in the univariate analysis (*P*=.45).

**Table 2:** Univariate regression result and correlation matrix for the variables of the study (N=40,505).

Variable	Univariate regression	Correlation matrix								
	logPAS^a^	1	2	3	4	5	6	7	8	9
1. Patient age	–0.00^b^	1								
2. Patient gender	0.04^b^	0.25^b^	1							
3. Patients’ waiting time for response	–0.21^b^	–0.02^b^	0.03^b^	1						
4. Patients’ initiation of consultation	0.92^b^	–0.03^b^	–0.02^b^	0.06^b^	1					
5. Patient cost for consultation	0.49^b^	0.02^b^	–0.04^b^	–0.08^b^	0.11^b^	1				
6. Physician age	0.00	0.07^b^	0.04^b^	0.12^b^	0.03^b^	0.23^b^	1			
7. Physician gender	0.30^b^	0.03^b^	0.26^b^	0.01^c^	0.03^b^	–0.02^c^	0.01	1		
8. Physician online consultation volume	–0.08^b^	–0.03^b^	0.06^b^	0.00	0.02^b^	0.30^b^	0.39^b^	0.07^b^	1	
9. Physician online consultation fee	0.01^b^	–0.06^b^	–0.05^b^	0.05^b^	0.06^b^	0.46^b^	0.34^b^	–0.06^b^	0.52^b^	1

^a^PAS: patient activeness score.

^b^*P*<.001.

^c^*P*<.01.

### Multilevel Models

Two-level regression analyses using maximum likelihood estimation were conducted to model how patient- and physician-level factors were associated with patient activeness in online medical consultations. In Table 3, the full model includes first-level factors, such as patient demographics and consultation behavioral factors. Specifically, these consultation behavioral factors were considered explanatory variables with random slopes at the patient level, controlling for age and gender. To simplify random-slopes models, correlations between intercepts and slopes were removed by assuming that the random effects (intercepts and slopes) are independent. All first-level factors were significantly associated with patient activeness. Patients’ waiting time for response (*β*=–.17; *P*<.001) during consultations showed a negative association with patient activeness, whereas patients’ initiation of consultation (*β*=.83; *P*<.001) and patient cost for consultation (*β*=.52; *P*<.001) were positively associated with activeness.

In addition, all physician-related variables except age showed a substantial effect. In terms of gender, patients communicating with female (*β*=.09; *P*=.01) physicians scored higher on the activeness measure. Physicians’ online consultation volume (*β*=–.10; *P*=.01) was negatively associated with patient activeness, whereas physician online consultation fee (*β*=.03; *P*=.01) was positively associated with patient activeness.

Also shown in Table 3 are the estimates from the full model with cross-level interactions between patients and physicians. The deviance difference test produced a chi-square of 15.5 (*df*=3; *P*=.001), indicating that the full model should be preferred compared with Model B without interactions (Multimedia Appendix 1). All the 3 proposed interactions were statistically significant at .05. The coefficient for the interaction between patients’ waiting time for response and physician online consultation volume was 0.05. The coefficient for the interaction between patients’ initiation of consultation and physician gender was –0.08, and the coefficients for the interaction between patient cost for consultation service and physician online consultation fee was 0.03.

**Figure 1:**
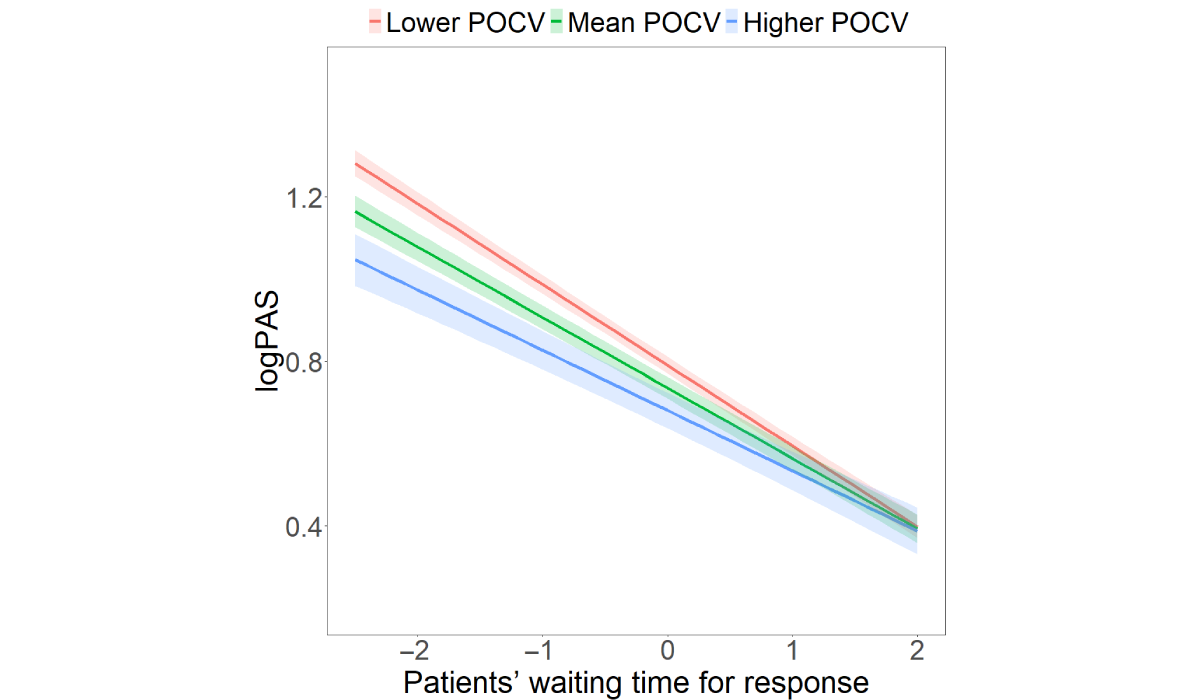
Interaction effects of patients’ waiting time for response and physician online consultation volumes (POCVs) on patient activeness. Note: Mean POCV is the average value of physicians’ online consultation volume. Higher and lower POCV were calculated by taking the mean POCV ± its SD.

To better illustrate the interactive effect between patients’ waiting time for response and physician online consultation volumes on patient activeness, physician online consultation volume was categorized into 3 categories: low, medium, and high. Figure 1 unveils that the negative relationship between patients’ waiting time for response and patient activeness was negatively moderated by physicians’ online consultation volumes (*β*=.05; *P*=.01). The longer patients waited for response, the less likely they were to actively participate in online medical consultation. In addition, patients tended to be even less active if their physicians had larger online consultations volumes than those with fewer volumes.

**Figure 2:**
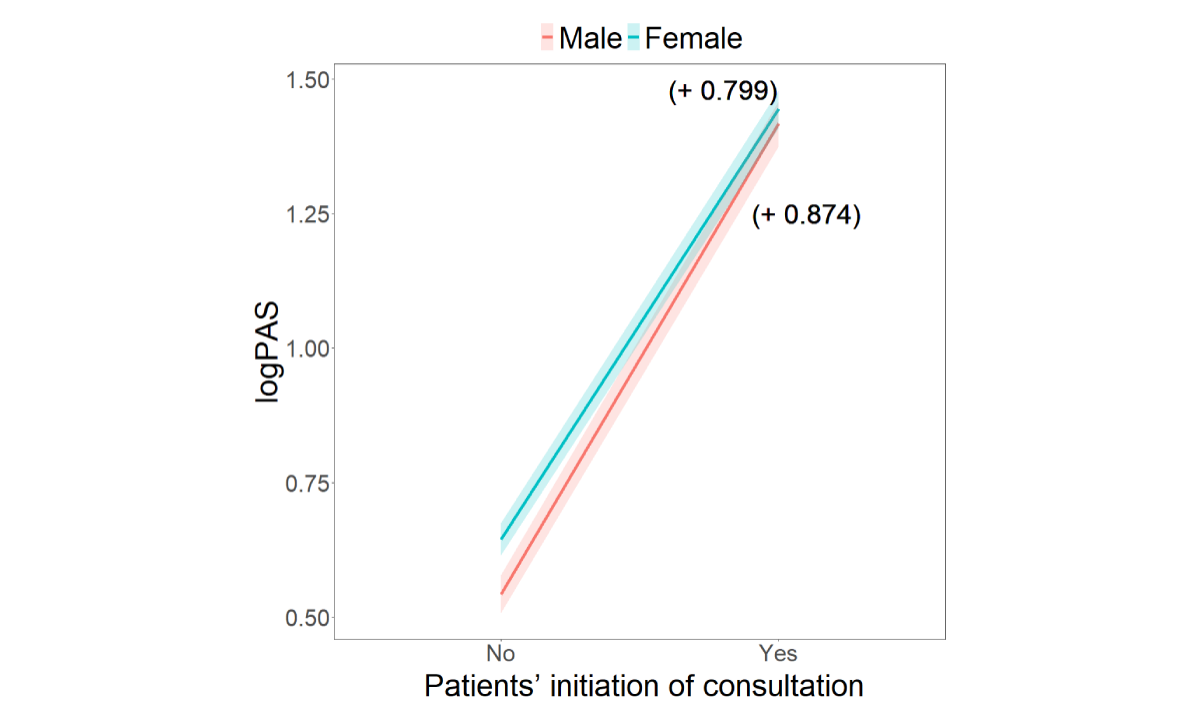
Interaction effects of patients’ initiation of consultation and physician gender on patient activeness.

In the interaction between patients’ initiation of consultation and physician gender, Figure 2 illustrates that the relationship between patients’ initiation of consultation and patient activeness was negatively moderated by physician gender (*β*=–.08; *P*=.03). Patient activeness was substantially higher during online medical consultations initiated by patient themselves than by the physician. Particularly, when patients initiated the conversation, logPAS increased by 0.799 in the female physician condition and by 0.874 in the male physician condition. This interactive effect also suggests that when physicians initiated the conversation after the consultation has been launched, female physicians are likely to encourage patients to participate more actively than male physicians.

**Figure 3:**
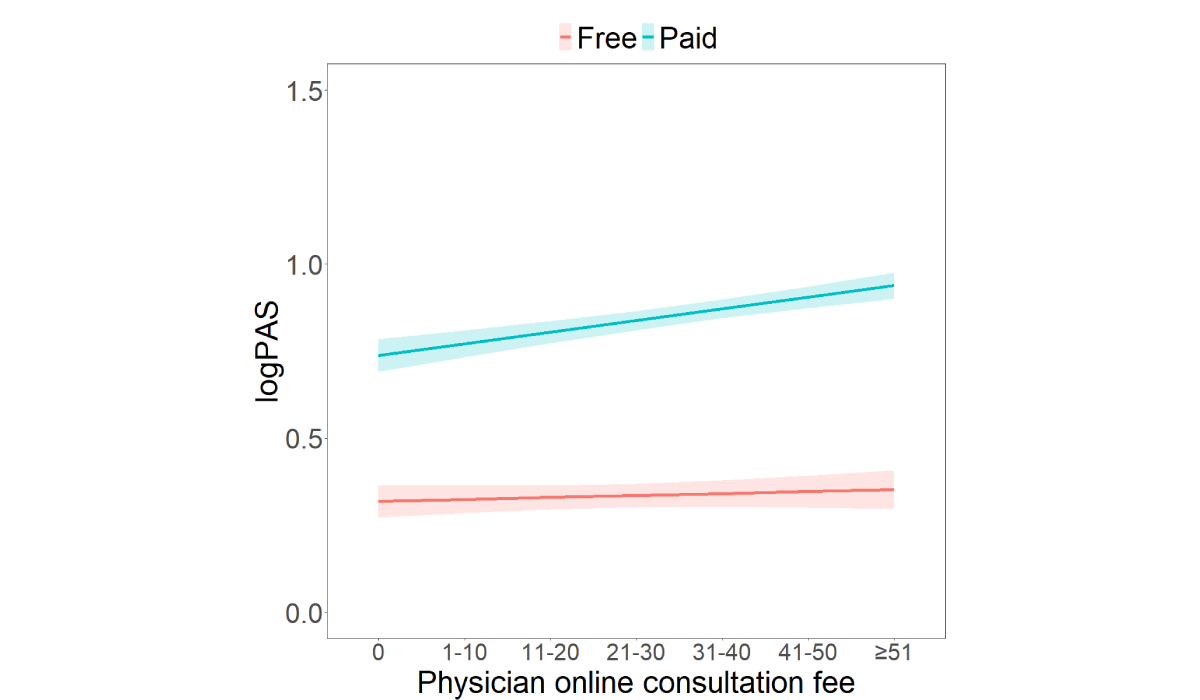
Interaction effects of patient cost for consultation and physician online consultation fee on patient activeness.

Figure 3 illustrates that the positive relationship between patient cost for consultation and patient activeness was positively moderated by physician online consultation fee (*β*=.03; *P*=.03). Patients who paid for their consultations were more likely to participate during online medical consultation. A higher physician online consultation fee was associated with more patient activeness from the paid patient.

**Table 3:** Multilevel models for patient activeness with individual- and physician-level factors (N=40,505 patients and 300 physicians).

Model			Full model
Variable			Coefficient (standard error)
Intercept			0.75 (0.10)^a^
**Patient level**			
	Patient age	–0.00 (0.00)^a^	
	Patient gender	0.06 (0.01)^a^	
	Patients’ waiting time for response	–0.17 (0.01)^a^	
	Patients’ initiation of consultation	0.83 (0.02)^a^	
	Patient cost for consultation service	0.52 (0.03)^a^	
**Physician level**			
	Physician age	0.00 (0.00)	
	Physician gender	0.09 (0.04)^c^	
	Physician online consultation volume	–0.10 (0.04)^b^	
	Physician online consultation fee	0.03 (0.01)^c^	
**Cross-level interaction**			
	Patients’ waiting time for response × physician online consultation volume	0.05 (0.02)^c^	
	Patients’ initiation of consultation × physician gender	–0.08 (0.03)^c^	
	Patient cost for consultation service × physician online consultation fee	0.03 (0.01)^c^	
	Akaike information criterion	101,969.6	
	Deviance	101,933.6	

^a^*P*<.001.

^b^*P*<.01.

^c^*P*<.05.

## Discussion

### Principal Findings

This study adopted a multilevel analysis to uncover factors associated with patient activeness in online medical consultation. Using a data set of 40,505 patients from 300 physicians in 10 specialties, this study found that patients were not equally active when participating in online medical consultation. Patient characteristics, consultation practices, and physician professional attributes are associated with patient activeness, such that (1) young and female patients tended to be more active in online medical consultation; (2) short waiting times, high cost, and patients’ initiation of conversation were associated with higher patient activeness; (3) patients consulting with female physicians and physicians with relatively low online consultation volumes were more likely to actively participate in online medical consultation. The disparities in patient activeness do not only result from patient autonomy, but also from the interaction between physician professional practices and online consultation contexts. This study has implications for other low- and middle-income countries where medical resources are limited and hospital burdens are high.

This study found that the characteristics of the patients may be associated with their activeness. The age of the patients is associated with their activeness, echoing previous studies that found older patients were fearful or less confident in the “digital world” [23]. Older patients are more likely to believe and follow physicians’ instructions, given that they often view doctors as responsible for medical decisions rather than themselves [24]. Furthermore, this study found that female patients were more likely to participate in online medical consultations. These results are consistent with other gender studies in the health care field, such that men are frequently underengaged with medical decision making due to masculinity concerns [23] and poor communication skills [25]. Nevertheless, we could not conclude that men and older patients are not necessarily incapable or unwilling to participate in online medical consultation. Given the advantages of patient activeness in shared medical decision making and treatment adherence [26], more men and older patients should be encouraged to actively participate during consultation.

In addition, this study suggests that patients’ waiting time for response, initiation of conversation, and service cost were significant factors influencing patient activeness in online medical consultation. Previous studies have shown a negative relationship between waiting time and patient satisfaction in the offline settings [27], and the ability to save time is regarded as the most evident benefit for patients using online medical consultation services [28]. However, this study reveals that patients’ median waiting time for an online physician response is approximately 2 hours in China, which is longer than most studies conducted in offline settings [27,29]. This challenges the naïve idea that online medical consultation typically saves time for patients. Given that most online medical consultation transpires in an asynchronous and discontinuous manner, online medical consultation could cost more time. Under this circumstance, it is important to recognize that responding timely becomes an even crucial factor for both patient activeness and patient satisfaction [1]. Moreover, patients’ initiation of conversation in online medical consultation was positively associated with their activeness. In a traditional medical setting, physicians tend to be in the dominant position, initiating the conversation. Online communication grants patients the option to initiate the communication and indicate personal preferences. Patients who took the initiative to break the silence or make greetings demonstrate positive intentions for establishing rapport with subsequent interactions, facilitating possible joint decision making [26]. Last but not least, cost for consultations is also associated with patient activeness. In contrast to free patients, patients who paid for consultation services were more likely to participate, given that their perceived input was higher and they expected better outcomes [30]. The patients who paid higher fees might be more motivated to actively participate in the interaction with physicians to maximize the high cost and make better medical decisions.

The study also shows that a physician’s demographics and professional characteristics are associated with patient activeness. Patients who communicated with female doctors, instead of male doctors, were likely to be more active in online medical consultation. There have been several qualitative studies on how a physician’s communication efforts can establish understanding and rapport with patients and encourage patient activeness [31]. Women physicians tend to have longer consultation time, engage in more partnership building, and are more interested in psychosocial aspects of health [25], allowing patients to become more engaged during consultation. Moreover, physicians with high online consultation volumes tended to discourage patients from participating more actively. It is a dilemma that many patients prefer to seek care from experienced chief physicians who have treated a large number of patients in China [32], but the chief physicians might not have enough time to interact with their specific patient because of the volume of patients. Patients lose their enthusiasm for active participation if they wait long for a physician with a large number of online consultations, as they may understand the physician’s workload and expect less attention from him/her [33]. These findings validate the gender effect of physicians and suggest that the dynamics of patient-physician interactions are closely related to patient behaviors. Physicians are encouraged to play an integral part in increasing patient activeness during online consultation process.

### Limitations

This study is not without limitations. Because of limited patient characteristics in our data set, we failed to identify more personal factors that may be associated with patient activeness. Furthermore, despite the high number of patients involved in this study, only 300 physicians from 10 different specialties were included. To verify current findings, future studies with a larger pool of physicians are required. In addition, text analysis can be synchronously conducted along with this study design to closely examine the interactive dynamics between patients and physicians during online medical consultation.

### Conclusion

Relying on a multilevel analysis of 40,505 patients and 300 physicians, this study is among the early studies that identified a triangular model related to patient activeness in online medical consultation. The triangular factors include patient characteristics, consultation behavioral attributes, and physician professional characteristics. This study suggests that reducing patients’ online waiting time and encouraging patients’ initiation of consultation are related to the increase of patient activeness in low- and middle-income countries. The findings of this study have practical implications for expanding patient-centered services and improving patient experiences with online consultation services to reduce the pressure and burden of offline medical services.

## Appendix

Multimedia Appendix 1 Multilevel models for patient activeness with individual- and physician-level factors (N=40505, 300 physicians).

## Abbreviations

ICC: intraclass correlation coefficient
PAS: patient activeness score
