# Supplementary material for: Patient Activeness During Online Medical Consultation in China: Multilevel Analysis
Source: J Med Internet Res. 2022 May 27;24(5):e35557. doi: 10.2196/35557 (PMC9187968; doi:10.2196/35557)
Supplement: Multimedia Appendix 1 [file jmir_v24i5e35557_app1.docx]

Multimedia Appendix 1**.** Multilevel models for patient activeness with individual- and physician-level factors (N=40505, 300 physicians)

| Model | Null model | Model A | Model B | Full model |
| --- | --- | --- | --- | --- |
| Variable | Coefficient (s.e.) | Coefficient  (s.e.) | Coefficient  (s.e.) | Coefficient  (s.e.) |
| Intercept | .69 (.02)^a^ | .83 (.02)^a^ | .76 (.10)^a^ | .75 (.10)^a^ |
| **Patient level** |  |  |  |  |
| Patient age |  | −.00 (.00)^a^ | −.00 (.00)^a^ | −.00 (.00)^a^ |
| Patient gender |  | .06 (.01)^a^ | .06 (.01)^a^ | .06 (.01)^a^ |
| Patients’ waiting time for response |  | −.19 (.01)^a^ | −.19 (.01)^a^ | −.17 (.01)^a^ |
| Patients’ initiation of consultation |  | .83 (.02)^a^ | .83 (.02)^a^ | .83 (.02)^a^ |
| Patient cost for consultation service |  | .49 (.02)^a^ | .49 (.02)^a^ | .52 (.03)^a^ |
| **Physician level** |  |  |  |  |
| Physician age |  |  | .00 (.00) | .00 (.00) |
| Physician gender |  |  | .09 (.04)^c^ | .09 (.04)^c^ |
| Physician online consultation volume |  |  | −.10 (.04)^b^ | −.10 (.04)^b^ |
| Physician online consultation fee |  |  | .02 (.01)^c^ | .03 (.01)^b^ |
| **Cross-level interaction** |  |  |  |  |
| Patients’ waiting time for response  × Physician online consultation volume |  |  |  | .05 (.02)^c^ |
| Patients’ initiation of consultation  × Physician gender |  |  |  | −.08 (.03)^c^ |
| Patient cost for consultation service  × Physician online consultation fee |  |  |  | .03 (.01)^c^ |
| **AIC** | 108980.9 | 101984.3 | 101979.1 | 101969.6 |
| **Deviance** | 108974.9 | 101962.3 | 101949.1 | 101933.6 |

^a^ *P*＜ .001; ^b^ *P*＜ .01; ^c^ *P*＜ .05
